# A review on quinoline derivatives as anti-methicillin resistant *Staphylococcus aureus* (MRSA) agents

**DOI:** 10.1186/s13065-020-00669-3

**Published:** 2020-03-13

**Authors:** Pradeep Kumar

**Affiliations:** Department of Pharmaceutical Sciences and Natural Products, Central University of Punjab, Bathinda, 151001 India

**Keywords:** *Staphylococcus aureus*, MRSA, Quinoline, CA-MRSA, HA-MRSA, LA-MRSA

## Abstract

Methicillin Resistant *Staphylococcus aureus* (MRSA) consists of strains of *S. aureus* which are resistant to methicillin. The resistance is due to the acquisition of mecA gene which encodes PBP2a unlike of any PBPs normally produced by *S. aureus*. PBP2a shows unusually low β-Lactam affinity and remains active to allow cell wall synthesis at normally lethal β-Lactam concentrations. MRSA can cause different types of infections like Healthcare associated MRSA, Community associated MRSA and Livestock associated MRSA infections. It causes skin lesions, osteomyelitis, endocarditis and furunculosis. To treat MRSA infections, only a few options are available like vancomycin, clindamycin, co-trimoxazole, fluoroquinolones or minocycline and there is a dire need of discovering new antibacterial agents that can effectively treat MRSA infections. In the current review, an attempt has been made to compile the data of quinoline derivatives possessing anti-MRSA potential reported to date.
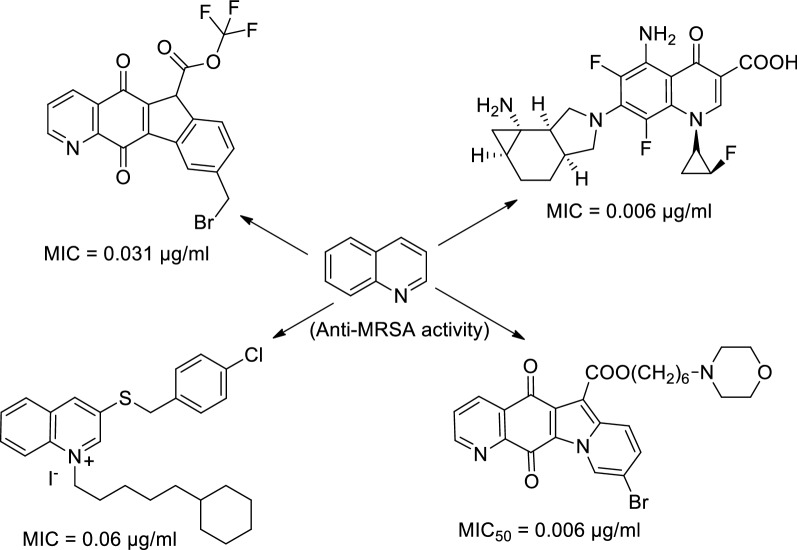

## Introduction

*Staphylococcus aureus*, a Gram-positive bacterium, is a member of the family Micrococcaceae, whose cells tend to occur either singly or if dividing cells do not separate, form pairs, tetrads and distinctive irregular “grape-like” structures [1]. *Staphylococcus aureus* is a very important bacterium because it can cause a wide range of diseases such as rashes, inflammations of bones and the meninges as well as septicemia and has a capacity to adapt to different environments [2].

### Prevalence of methicillin resistant *Staphylococcus aureus* (MRSA)

In 1965, MRSA infection in Australia was first recorded in Sydney. First case of hospital MRSA in the United States was reported in Boston in 1968. The first methicillin strain of *S. aureus* was identified in Europe in the United Kingdom. Till 1970s, MRSA infections in Europe were limited to hospital outbreaks. In Japan, MRSA was detected for first time in 2003. In 2011, China showed a mean MRSA rate of 45.8% among all clinical *S. aureus* isolates [3].

India is also not an exception in this aspect and high prevalence of MRSA is an emerging health problem. MRSA prevalence in India has significantly increased from 12% in 1992 to 40% in 2009 [4] with minimum incidence of 25% in western India and maximum of 50% in South India [5]. Bouchiat et al. Found that 54.8% of the total *S. aureus* isolates among samples from a hospital in eastern Uttar Pradesh were methicillin resistant. Further, 57.3% of the blood cultures from a Neonatal Intensive Care Unit in Amritsar were methicillin resistant [6].

MRSA infection may be of three types i.e. Healthcare associated MRSA (HA-MRSA), Community associated MRSA (CA-MRSA) and Livestock associated MRSA (LA-MRSA). Vysakh and Jeya performed a comparative analysis of community acquired and hospital acquired methicillin resistant *Staphylococcus aureus* on 450 *Staphylococcus aureus* isolates and found that out of 450 isolates, 121 (27%) were methicillin resistant *Staphylococcus aureus* (MRSA) containing 91 (20%) HA-MRSA and 30 (7%) CA-MRSA [7].

Kali et al. studied resistance pattern of methicillin resistant *Staphylococcus aureus* on one hundred two clinical isolates of MRSA and found that MRSA isolates showed high resistance to co-trimoxazole (82.3%), ciprofloxacin (76.4%), gentamicin (64.7%) and tetracycline (49%) as compared to other drugs [4].

#### Healthcare associated MRSA (HA-MRSA)

HA-MRSA means MRSA isolates from hospitals and are gradually increasing round the globe. The rate of HA-MRSA infections is high (> 50%) in USA, Asia and Malta. Asian countries like South Korea (77.6%), Vietnam (74.1%), Taiwan (65%) and Hong Kong (56.8%) have higher incidence of HA-MRSA infections. Intermediate rate (25–50%) is reported in Africa, China and Europe. In HA-MRSA, anterior nare is the usual site for MRSA colonization. Hands, perineal region, skin wounds, throat, genitourinary tract and the digestive tract may also colonize MRSA. Generally, HA-MRSA results in dermatitis, septicemias, heart and lung diseases. Risk factors for HA-MRSA include hospitalization, surgery, dialysis and previous history of MRSA infection [2].

#### Community associated MRSA (CA-MRSA)

Community associated means MRSA isolates from community living away from hospital settings. In 1993, first case of CA-MRSA was reported in Western Australia. Military personnel, prison inmates, athletes, intravenous drug users are at high risk for CA-MRSA. Elderly, children, patients having implanted medical devices, people suffering from diseases like diabetes or neutrophil dysfunction, HIV/AIDS and influenza are also at high risk for CA-MRSA infection [8].

#### Livestock associated MRSA (LA-MRSA)

MRSA was considered as a human infection until when it was isolated in a dairy cow with mastitis and in pigs [2]. MRSA can transfer from humen to animals and vice versa. Voss et al. reported 23% of pig farmers infected with MRSA from a pig farm in the Netherlands [9] and VanRijen et al. found 32% of farm workers colonized with MRSA [10]. This overcoming of the genus barrier by LA-MRSA strains indicates its host adaptability and shows that livestock animals can serve as a reservoir for infections in humen [8].

## Main text

### Mechanism of resistance

β-Lactam antibiotics inhibit penicillin binding proteins which lead to weakened cell wall and ultimately the cell death. Methicillin resistance in the MRSA strain is due to the acquisition of *mec* element and *mec*A gene which encodes PBP2a unlike of any PBPs normally produced by *S. aureus*; *mec* element codes for recombinase proteins causing excision and integration of *mec* element in bacterial chromosome. MecI and MecR1 proteins regulate the synthesis of PBP2a, former being a signal transduction protein and later is a transcription regulator [1]. PBP2a shows unusually low β-Lactam affinity and remains active to allow cell wall synthesis at normally lethal β-Lactam concentrations [11].

### Quinoline derivatives as anti-MRSA agents

Quinoline is a versatile heterocyclic moiety having diverse spectrum of biological activities including anti- Alzheimer’s [12], anticancer [13], anticonvulsant [14], antidiabetic [15], antihypertensive [16], anti-inflammatory [17], antimicrobial [18] and ubiquitination inhibition [19] activities. In this review we hereby report anti-MRSA activity of quinoline derivatives reported so far.

Fu et al. synthesized a series of quinoline derivatives as broad-spectrum antibacterial agents. The antibacterial activity of the synthesized compounds against panel of bacterial strains including resistant strains. The synthesized compounds displayed good antibacterial activity against both Gram negative and positive bacterial strains. Compound **1** (a derivative of ciprofloxacin) emerged as most potent antibacterial agent of the series having MIC value 8 µg/mL against MRSA strain ATCC33591 (Table 1) [20].

**Table 1:** Anti-MRSA activity of compound **1**

Compound	Structure	MIC (µg/mL)
**1**		8
Ciprofloxacin	**–**	0.25

Zheng et al. carried out antibacterial evaluation of a series of benzofuroquinolinium derivatives as FtsZ polymerization inhibitors which inhibits cell division and causes cell death. All the five compounds were tested against a panel of bacterial strains including resistant strains. Among the tested compounds, **2** was most potent anti-MRSA agent having MIC values 1, 1, 1, 1 and 0.5 µg/mL against MRSA strains ATCC 43300, BAA-41, 33591, BAA-1720 and 33592 respectively and exerted its effect by inhibiting cell-division protein FtsZ (Table 2) [21].

**Table 2:** Anti-MRSA activity of compound **2**

Compound	Structure	MIC (µg/mL)				
		ATCC-43300	BAA41	ATCC-33591	BAA-1720	ATCC-33592
**2**		1	1	1	1	0.5
Methicillin		512	1024	1024	1024	512

Teng et al. synthesized a series of quinoline derivatives as antibacterial agents. The synthesized compounds were tested for their antibacterial activity against resistant bacterial strains viz. MRSA, MRSE and VRE. Antibacterial activity results indicated that compound **3** was most potent anti-MRSA agent having MIC value 1.5 µg/mL (Table 3) [22].

**Table 3:** Anti-MRSA activity of compound **3**

Compound	Structure	MIC (µg/mL)
**3**		1.5
Daptomycin		0.5

Sun et al. synthesized a series of *N*-methylbenzofuro[3,2-b]quinoline and *N*-methylbenzoindolo[3,2-b]-quinoline derivatives and evaluated it’s in vitro antibacterial activity against *B. subtilis* 168, *S. aureus* ATCC 29213, *S. aureus* ATCC BAA41 (MRSA), *E. faecium* ATCC 49624, *E. faecium* ATCC 700221 (vancomycin-resistant strain), *E. coli* ATCC 25922, *E. coli* ATCC BAA2469 (expressing NDM-1 beta-lactamase), *P. aeruginosa* ATCC BAA2108 (multidrug-resistant strain) and *K. pneumonia* ATCC BAA2470 (expressing NDM-1 beta-lactamase). Antibacterial acivity results indicated that compounds **4**–**6** (MIC = 2 µg/mL) were found to be most potent antibacterial agents and were almost 100 times more potent than standard drugs berberine (MIC = 192 µg/mL) and methicillin (MIC > 192 µg/mL). These compounds inhibited the GTPase activity and polymerization of FtsZ causing abnormal cell division and cell death (Table 4) [23].

**Table 4:** Anti-MRSA activity of compounds **4**–**6**

	Comp	Structure	Comp	Structure	Comp	Structure	Comp	
	**4**		**5**		**6**		Berberine	Methicillin
MIC (µg/mL)	2		2		2		192	> 192

Yang et al. synthesized a series of 9-bromo substituted indolizinoquinoline-5,12-dione derivatives and screened its antibacterial activity against gram-negative and gram-positive bacterial strains including 100 clinical MRSA strains. Antibacterial evaluation results indicated that the synthesized compounds displayed good antibacterial activity and compound **7** (6-Morpholinohexyl 9-bromo-5,12-dioxo-5,12-dihydroindolizino[2,3-g]quinolone-6-carboxylate) was found to be most potent antibacterial agent against clinical MRSA strains having both MIC_50_ and MIC_90_ values lower than 7.8 ng/mL. Compound **8** (6-(Piperidin-1-yl)hexyl-9-bromo-5,12-dioxo-5,12-dihydro-indolizino[2,3-g]quinoline-6-carboxylate), having water solubility of 1.98 mg/mL was also found to be very potent against clinical MRSA strains with MIC_50_ value of 63 ng/mL and MIC_90_ value of 125 ng/mL, 16-fold higher than that of vancomycin (MIC_50_ = 1 µg/mL and MIC_90_ = 2 µg/mL) (Table 5) [24].

**Table 5:** Anti-MRSA activity of compounds **7**–**8**

	Comp	Structure	Comp	Structure	Comp
	**7**		**8**		Vancomycin
MIC_50_ (ng/mL)	< 7.8		63		1 µg/mL
MIC_90_ (ng/mL)	< 7.8		125		2 µg/mL

Challa et al. synthesized series of indolo[2,3-b]quinolines, Chromeno[2,3-b]indoles, and 3-Alkenyl-oxindoles from 3,3′-Diindolylmethanes. The synthesized compounds were tested in vitro for their antibacterial activity against a panel of Gram positive and negative bacterial strains including MRSA. The results indicated that compound **9** was found to be most potent anti-MRSA agent having MIC value 2 µg/mL against MRSA strain MRSA-ATCC33591 and 1 µg/mL against MRSA-R3545, MRSA-R3889 and MRSA-R3890 (Table 6) [25].

**Table 6:** Anti-MRSA activity of compound **9**

Compound	Structure	MIC (µg/mL)			
		ATCC-33591	R3545	R3889	R3890
**9**		2	1	1	1
Methicillin	–	> 64	32	32	> 64

Dolan et al. reported the synthesis and antibacterial evaluation of a series of thiourea-containing compounds. All the synthesized compounds were evaluated for their bacteriostatic activity against *E. coli, P. aeruginosa* and *S. aureus.* Antibacterial activity results indicated that compound **10** (1-(3,5-Bis(trifluoromethyl)phenyl)-3-((S)-(6-methoxyquinolin-4-yl)-((1S,2S,4S,5R)-5-vinylquinuclidin-2-yl)methyl)thiourea), was the most active antibacterial agent and exhibited bacteriostatic activity against methicillin resistant *Staphylococcus aureus* (MRSA, MIC_50_ = 11.44 µM, MIC_90_ = 17.74 µM) (Table 7). Compound **10** was also evaluated for its in vivo toxicity using the larvae of the Greater wax moth, *Galleria mellonella* and was found to be non-toxic to the larvae of *Galleria mellonella* up to 1000 µg/mL [26].

**Table 7:** Anti-MRSA activity of compound **10**

Compound	Structure	MIC_50_ (µg/mL)	MIC_90_ (µg/mL)
**10**		11.44	17.74

Perkovic et al. designed and synthesized a series of novel compounds with primaquine and hydroxyl or halogen substituted benzene moieties bridged by urea or bis-urea moiety using benzotriazole as the synthon. The synthesized compounds were tested in vitro for their antimicrobial activity against a panel of 15 bacterial strains and a fungal strain, *C. albicans* using primaquine and tetracycline hydrochloride (TC) or voriconazole (VOR) as standard drugs. Antimicrobial activity results showed that only four compounds (**11**–**14**) were having good antibacterial/antifungal effect. The most potent compound, **14** was having MIC value ranged from 1.6 to 12.5 µg/mL against the selected bacterial strains including MSSA [27].

Takahashi et al. examined effect of indolo[3,2-*b*]quinoline derivatives on hemolysis induced by the aerolysin-like hemolysin (ALH) of *Aeromonas sobria* and also by the alpha hemolysin of *Staphylococcus aureus.* They observed that hemolysis induced by ALH was significantly reduced by all four derivatives while alpha mediated hemolysis was significantly reduced by three of them. Compounds **15** and **16** having amino group at the C-11 position of indolo[3,2-b]quinoline, showed strong ALH inhibitory activity and compound **17** consisting benzofuran and quinoline displayed strong alpha-hemolysin inhibitory effects [28].

**Table:** 

		
**11**	**12**	**13**
		
**14**	**15**	**16**

Wang et al. synthesized a series of 2-phenyl-quinoline-4-carboxylic acid derivatives and evaluated their antibacterial activities against *Escherichia coli, Pseudomonas aeruginosa*, *Staphylococcus aureus, Bacillus subtilis* and one strain of methicillin-resistant *Staphylococcus aureus* (MRSA) by the agar diffusion and broth dilution method, which indicated that compound **18** was most potent compound against MRSA having zone of inhibition 5 ± 0.5 and 6 ± 0.2 mm at 50 and 100 µg/mL respectively. Further, MTT assay displayed the low cytotoxicity of compound **18** [29].

**Table:** 

	
**17**	**18**

Zhang et al. synthesized a series of benzimidazole quinolones as potential antimicrobial agents. The synthesized compounds were screened for their antimicrobial activity against a group of Gram positive and Gram negative bacterial strains. The compounds were designed with an aim to overcome the resistance against quinolones. Literature studies reveal that C-7 position of quinolones is in closed proximity to the Arg456 in the topoIV-DNA complex, mutation in which leads to resistance making the quinolone C-7 position a site of strategic importance in overcoming the resistance. Moreover substituents at C-7 influence the cell permeability and effect the bacterial resistance. In order to modify quinolone C-7 position, benzimidazole moiety was introduced. Antibacterial activity results showed that compounds **19**–**22** were found to be the most potent antibacterial agents against MRSA, each having MIC value 0.125 µg/mL (Table 8). Compounds **19**–**22** intercalated DNA by forming complex with it and blocked its replication especially by interaction with Ser79 [30].

**Table 8:** Anti-MRSA activity of compounds **19**–**22**

Comp	Structure	MIC (µg/mL)	Comp	Structure	MIC (µg/mL)
**19**		0.125	**20**		0.125
**21**		0.125	**22**		0.125
Clinafloxacin		1	Ciprofloxacin		2

Zhao et al. synthesized a series of indoloquinoline analogs including Indolo[3,2-b]quinolone, 4-(acridin-9-ylamino) phenol hydrochloride, benzofuro[3,2-b]quinoline and indeno[1,2-b]quinolones. The synthesized compounds were screened for their anti-MRSA activity against OM481 and OM584 strains. Results indicated that Indolo[3,2-b]quinoline analog (**23**) and benzofuro[3,2-b]quinoline (**24**) were most potent anti-MRSA agents, both having MIC values 2 µg/mL against both MRSA strains (Table 9) [31].

**Table 9:** Anti-MRSA activity of compounds **23**–**24**

Comp	Structure	MIC (µg/mL)		Comp	Structure	MIC (µg/mL)	
		OM481	OM584			OM481	OM584
**23**		2	**2**	**24**		2	2
Vancomycin		2	1				

Huang et al. synthesized a series of levofloxacin core-based derivatives and were screened them for their antimicrobial activity against selected bacterial strains. Antibacterial screening results indicated that compounds **25**–**28** were most potent anti-MRSA agents each having MIC value 1 µg/mL (Table 10) [32].

**Table 10:** Anti-MRSA activity of compounds **25**–**28**

Comp	Structure	MIC (µg/mL)	Comp	Structure	MIC (µg/mL)
**25**		1	**26**		1
**27**		1	**28**		1
Vancomycin		1	Moxifloxacin		4

Zhang et al. designed and synthesized a series of fluoroquinolone derivatives containing 3-alkoxyimino-4-(cyclopropylanimo)methylpyrrolidine moiety and evaluated their antibacterial activity against a panel of Gram-negative and Gram-positive strains. The antibacterial activity results indicated that compound **29** was found to be most potent MRSA inhibitor having MIC values of 2 µg/mL against both MRSA 14-4 and 14-5 strains (Table 11) [33].

**Table 11:** Anti-MRSA activity of compound **29**

Comp	Structure	MIC (µg/mL)		Comp	MIC (µg/mL)	
		MRSA 14-4	MRSA 14-5		MRSA 14-4	MRSA 14-5
**29**		2	**2**	Levofloxacin	64	8

Cui et al. designed and synthesized a novel series of hybrids of metronidazole and quinolones and evaluated their antibacterial activities against Gram-positive bacteria (*S. aureus* ATCC 6538, Methicillin-resistant *Staphylococcus aureus* N315 (MRSA) and *B. subtilis* ATCC 21216), Gram-negative bacteria (*E. coli* ATCC 8099*, P. aeruginosa* ATCC 27853 and *B. proteus* ATCC 13315). The compounds were designed with an aim to overcome the resistance against quinolones. Literature studies reveal that N-1 position of quinolones is in closed proximity to the residues Ser79 and Asp83 in the topoIV-DNA complex, mutation in which leads to resistance making the quinolone N-1 position a site of strategic importance in overcoming the resistance. In order to modify quinolone N-1 position, nitroimidazole moiety was introduced based on their previous studies with a possibility of its non-covalent interactions with DNA base and thus overcoming the resistance. The antibacterial activity results indicated that compound **30** was most potent anti-MRSA agent with MIC value 1 µg/mL and was more potent than standard drugs chloromycin and norfloxacin (MIC values 8 and 2 µg/mL respectively, Table 12). Mechanism of action of compound **30** was investigated by studying its interactions with calf thymus DNA which showed non-covalent interaction between compound **30** and topo IV DNA complex, especially hydrogen bonds between compound **30** and Ser79 [34].

**Table 12:** Anti-MRSA activity of compound **30**

Comp	Structure	MIC (µg/mL)	Comp	MIC (µg/mL)	Comp	MIC (µg/mL)
**30**		1	Chloromycin	8	Norfloxacin	2

Abouelhassan et al. synthesized a series of quinoline derivatives having potent biofilm dispersal activity against methicillin-resistant *S. aureus.* They found that 9 out of 11 synthesized compounds were having better biofilm clearing activity than standard drug nitroxoline (Ec_50_ value 10.5 µM) and compound **31** was most potent and effective in clearing the biofilm established by MRSA-2 strain with an EC_50_ value 2.06 µM (Table 13) [35].

**Table 13:** Anti-MRSA activity of compound **31**

Comp	Structure	EC_50_ (µM)	Comp	EC_50_ (µM)
**31**		2.06	Nitroxoline	10.5

Cui et al. synthesized a novel series of quinolone triazoles and characterized it by using spectral techniques. The compounds were designed based on the results of their previous study indicating the importance of triazolyl ethanol moiety in the C-7 side chain of ciprofloxacin. Introduction of triazole ring at N-1 position of quinolones also improved their antimicrobial activity. Based on the improved antimicrobial activity of hybrid compounds containing quinolone and triazole moiety, triazolyl ethanol fragment into the N-1 position was incorporated. The synthesized compounds were screened for their antimicrobial activities against seven bacterial and four fungal strains including MRSA. The antibacterial activity results indicated that compound **32** was found to be most potent antibacterial agent against MRSA with an MIC value of 0.5 µg/mL (Table 14). Mechanism of action of compound **32** was investigated by studying its interactions with calf thymus DNA by fluorescence and UV–vis absorption spectroscopy results of which indicated that compound **32** intercalated DNA by forming complex with it and blocked its replication [36].

**Table 14:** Anti-MRSA activity of compound **32**

Comp	Structure	MIC (µg/mL)	Comp	MIC (µg/mL)	Comp	MIC (µg/mL)
**32**		0.5	Chloromycin	16	Norfloxacin	8

Guo et al. synthesized three series of rhodanine derivatives bearing a quinoline moiety and evaluated their antibacterial activity against a panel of Gram positive and Gram negative strains including clinical isolates of multidrug-resistant Gram-positive strains. The aim of the study was to modulate hydrophobicity of the rhodanine derivatives by changing the substituents at 3 and 5 positions and their effect on enzyme binding affinity. The compounds were designed based on the results of their previous study. The antibacterial activity results indicated that compounds **33**–**36** were most potent anti-MRSA agents, each having MIC value 1 µg/mL against both MRSA 3167 and 3506 strains and were equipotent to standard drug moxifloxacin and more potent than norfloxacin, gatifloxacin and oxacilin (Table 15) [37].

**Table 15:** Anti-MRSA activity of compounds **33**–**36**

Comp	Structure	MIC (µg/mL)		Comp	MIC (µg/mL)	
		3167	3506		3167	3506
**33**		1	1	Norfloxacin	8	4
**34**		1	1	Moxifloxacin	1	1
**35**		1	1	Gatifloxacin	2	1
**36**		1	1	Oxacillin	> 64	> 64

Bolden Jr. et al. synthesized 3-substituted benzylthioquinolinium iodide derivatives and evaluated their antimicrobial activity against selected fungal strains including resistant strains (MRSA) and pathogenic opportunistic bacterial strains, results of which indicated that compound **37** was most potent anti-MRSA agent having IC_50_, MIC and MBC values 0.06, 0.16 and 0.31 µg/mL respectively (Table 16) [38].

**Table 16:** Anti-MRSA activity of compound **37**

Comp	Structure	IC_50_ (µg/mL)	MIC (µg/mL)	MBC (µg/mL)	Comp	IC_50_ (µg/mL)	MIC (µg/mL)	MBC (µg/mL)
**37**		0.06	0.16	0.31	Ciprofloxacin	0.11	0.5	1

Cieslik et al. synthesized a series of new ring-substituted styrylquinolines and two oxorhenium complexes. The synthesized compounds were screened for their antimicrobial potential against a panel of bacterial and fungal strains which indicated that compound **38** was the most potent anti-MRSA agent having MIC/IC_90_ value of 3.9 and 7.81 µmol/mL at 24 and 48 h respectively and was more potent to all standard drugs bacitracin, penicillin V and ciprofloxacin (Table 17) [39].

**Table 17:** Anti-MRSA activity of compound **38**

Comp	Structure	IC_90_ (µM/mL)		Comp	IC_90_ (µM/mL)	
		24 h	48 h		24 h	48 h
**38**		3.9	7.81	Bacitracin	15.62	31.62

Sevgi et al. synthesized novel glyoximes containing quinoline moiety. The antibacterial activity of synthesized compounds was tested against selected bacterial strains. The antibacterial activity results indicated that only compound **39** showed good anti-MRSA activity (zone of inhibition = 12 mm) and was equipotent with standard drugs Amoxicillin/Clavulanic acid and Gentamicin (Table 18) [40].

**Table 18:** Anti-MRSA activity of compound **39**

Comp	Structure	Inhibition zone (mm)	Comp	Inhibition zone (mm)	Comp	Inhibition zone (mm)
**39**		12	Amoxicillin/clavulanic acid	12	Gentamicin	12

Wu et al. synthesized a series of 9-bromo-substituted indolizinoquinoline-5,12-dione derivatives. The synthesized compounds were evaluated for their antimicrobial activity against representative bacterial and fungal strains. The antimicrobial activity results indicated that compound **40** was found to be most potent anti-MRSA agent (MIC = 0.031 µg/mL) (Table 19) [41].

**Table 19:** Anti-MRSA activity of compound **40**

Comp	Structure	MIC (µg/mL)	Comp	MIC (µg/mL)
**40**		0.031	Penicillin	> 64

Chai et al. synthesized a series of novel gatifloxacin derivatives and characterized the synthesized compounds by using spectral techniques like ^1^H NMR, ^13^C NMR, MS and HRMS. The synthesized compounds were screened in vitro for their antibacterial potential against representative strains including MRSA and MRSE (Methicillin resistant *Staphylococcus epidermidis*). The antibacertial activity results revealed that the synthesized compounds were less active than parent compound, gatifloxacin and only compound **41** (MIC = 0.125 µg/mL) was equipotent with gatifloxacin (Table 20) [42].

**Table 20:** Anti-MRSA activity of compound **41**

Comp	Structure	MIC (µg/mL)	Comp	MIC (µg/mL)
**41**		0.125	Gatifloxacin	0.125

O’Donnell et al. reported antibacterial activity of 60 synthetic and naturally occurring quinolines against six Gram positive and Gram negative strains including MRSA. The antibacterial activity results indicated that compound **42** was most potent anti-MRSA agent having MIC value 0.39 µg/mL, was more potent than standard drugs oxacillin, vancomycin and trimethoprim (Table 21). The compound **42** was found to be DNA topoisomerase IV inhibitor [43].

**Table 21:** Anti-MRSA activity of compound **42**

Comp	Structure	MIC (µg/mL)	Comp	MIC (µg/mL)
**42**		0.39	Vancomycin	0.79

Lv et al. synthesized several amphiphilic cationic quinine-derived compounds and evaluated their in vitro antibacterial activity against different Gram positive and negative bacterial strains including methicillin-resistant *Staphylococcus aureus.* Compound **43** was found to be the best anti-MRSA agent having MIC values ranging from 0.39 to 0.78 µg/mL and MBC value 1.56 µg/mL against tested MRSA strains (Table 22) [44].

**Table 22:** Anti-MRSA activity of compound **43**

Comp	Structure	MIC (µg/mL)	MBC (µg/mL)
**43**		0.39–0.78/1.56	0.79

Wiles et al. synthesized a series of 9*H*-isothiazolo[5,4-b]quinoline-3,4-diones (ITQs) having aromatic substituent at the 7-position, using palladium-catalyzed cross-coupling. The antibacterial activity of synthesized compounds was assessed against Gram-positive and Gram-negative organisms. Antibacterial activity results indicated that in general, the synthesized compounds were more effective against Gram-positive than Gram-negative bacterial strains. Compounds **44** and **45** were most potent anti-MRSA agents, both having MIC value 0.125 µg/mL (Table 23) [45].

**Table 23:** Anti-MRSA activity of compounds **44**–**45**

Comp	Structure	MIC (µg/mL)	Comp	Structure	MIC (µg/mL)
**44**		0.125	**45**		0.125
Gemifloxacin		2.000	Moxifloxacin		2.000

Mardenborough et al. synthesized several N-substituted quindolines to study the effect of N-alkylation on the antimicrobial activity of selected quindolines. The synthesized compounds were evaluated for their antimicrobial activity against selected bacterial and fungal strains including MRSA which indicated that compound **46** was most potent anti-MRSA agent having IC_50_ value of 2.0 µg/mL (Table 24) [46].

**Table 24:** Anti-MRSA activity of compound **46**

Comp	Structure	IC_50_ (µg/mL)	Comp	IC_50_ (µg/mL)
**46**		2.00	Ciprofloxacin	0.15

Inagaki et al. synthesized a series of novel 6-fluoro-1-[(1R,2S)-2-fluorocyclopropan-1-yl]-4-oxoquinoline-3-carboxylic acids bearing cyclopropane fused 2-amino-8-azabicyclo[4.3.0]nonan-8-yl substituents at the C-7 position and evaluated their antibacterial activity against representative Gram positive and negative bacterial strains. The antibacterial activity results indicated that compounds **47** and **48** were most potent anti-MRSA agents, both having MIC values 0.006 µg/mL and were more potent than all the standard compounds levofloxacin, moxifloxacin, vancomycin and linezolid (Table 25) [47].

**Table 25:** Anti-MRSA activity of compounds **47**–**48**

Comp	Structure	MIC (µg/mL)	Comp	Structure	MIC (µg/mL)
**47**		0.006	**48**		0.006
Vancomycin		0.39	Moxifloxacin		0.78

Hoemann et al, synthesized a series of 2-(1*H*-indol-3-yl)tetrahydroquinolines using hetero Diels–Alder reaction and evaluated their antibacterial activity against methicillin-resistant *Staphylococcus aureus* (MRSA) and vancomycin-resistant *Enterococcus faecium* (VRE). The antibacterial activity results indicated that compounds **49** and **50** were most potent anti-MRSA agents having MIC values < 0.39 and 0.31 µg/mL respectively (Table 26) [48].

**Table 26:** Anti-MRSA activity of compounds **49**–**50**

Comp	Structure	MIC (µg/mL)	Comp	Structure	MIC (µg/mL
**49**		< 0.39	**50**		0.31

Hoemann et al. discovered a novel series 2-(1*H*-indol-3-yl)quinolones as anti-methicillin-resistant *Staphylococcus aureus* (MRSA) agents from a combinatorial library. The synthesized compounds were having minimum inhibitory concentrations (MICs) < 1.0 mg/mL against MRSA. A structure activity relationship (SAR) study was conducted for the anti-MRSA activity of synthesized quinolones which indicated that compounds having chloro or methyl alcohol group at 4th position of quinoline ring were having best anti-MRSA activity. Presence of chloro group at 5th, 6th, 7th and 8th position of quinoline ring also improved anti-MRSA activity of synthesized compounds. Presence of halo groups (Cl, Br or F) at 5th or 6th positions of indole nucleus also improved the anti-MRSA activity of the synthesized compounds. Compound **51** ((2-(5-bromo-1*H*-indol-3-yl)-7-chloroquinolin-4-yl)methanol) was found to be the most potent compound of the series (Table 27) [49].

**Table 27:** Anti-MRSA activity of compound **51**

Comp	Structure	MIC (µg/mL)
**51**		0.31

### Conclusion

The literature reports reveal that quinoline derivatives have immense potential to control MRSA infection. Many compounds have shown anti-MRSA activity better than standard drugs that too with low toxicity but microbes are also having an evolutionary feature of resistance. We have very limited options to deal with these emerging resistant microbes. So, we have to keep our war against emerging resistant microbes on and also we have to check the abuse of available antibiotics. This review will definitely help the researchers working on development of novel anti-MRSA agents.

## Data Availability

The data and material provided in the current manuscript is taken from available literature published in peer reviewed journals and proper references have been given for the same. All the references cited are available on the internet.

## Abbreviations

ATCC: American Type Culture Collection
*B. subtilis*: *Bacillus subtilis*
*C. albicans*: *Candida albicans*
CA-MRSA: Community associated MRSA
*E. coli*: *Escherichia coli*
*E. faecium*: *Enterococcus faecium*
FtsZ: Filamenting temperature-sensitive mutant Z
GTPase: Guanosine triphosphatase
HA-MRSA: Healthcare associated MRSA
LA-MRSA: Livestock associated MRSA
MIC_50_: Minimum inhibitory concentration which inhibits 50% isolates of the species, tested
MIC_50_: Minimum inhibitory concentration which inhibits 90% isolates of the species, tested
MRSA: Methicillin resistant *Staphylococcus aureus*
MRSE: Methicillin-resistant *Staphylococcus epidermidis*
MTT: 3-(4,5-Dimethylthiazol-2-yl)-2,5-diphenyltetrazolium bromide
NDM-1: New Delhi metallo-beta-lactamase 1
*P. aeruginosa*: *Pseudomonas aeruginosa*
VRE: Vancomycin-resistant *enterococcus faecium*
